# Retraction: MicroRNA-505-5p functions as a tumor suppressor by targeting Cyclin-Dependent Kinase 5 in cervical cancer

**DOI:** 10.1042/BSR-20191221_RET

**Published:** 2021-04-14

**Keywords:** Cervical cancer, cell invasion, cell migration, cell proliferation, CDK5, miR-505-5p

This Retraction follows an Expression of Concern relating to this article previously published by Portland Press.

This article is being retracted from *Bioscience Reports* at the request of the Editor in Chief and the Editorial Board following receipt of a notification from a reader, alerting the Editorial Board to duplication in Figure 5F. The Editorial Office has also identified evidence of potential image manipulation in this Figure.

The authors were contacted regarding the concerns raised and provided a replacement Figure 5F, however given that the original image showed signs of potential manipulation the Editorial Board stands by the decision to retract. The authors do not agree that the images were deliberately manipulated, but agree to the retraction.

